# Supplementary material for: WordCommentsAnalyzer: A windows software tool for qualitative research
Source: F1000Res. 2018 Sep 4;7:536. Originally published 2018 May 3. [Version 2] doi: 10.12688/f1000research.14819.2 (PMC6137411; doi:10.12688/f1000research.14819.2)
Supplement: Supplementary file 3 [file f1000research-7-17614-s0002.tgz › b2a79496-4ada-47c4-9682-a99ce867836d.zip!/Eight Iranian Health Journals/Archives of Rehabilitation_31.docx]

© Archives of Rehabilitation. This work is licensed under CC BY-NC 4.0 (https://creativecommons.org/licenses/by-nc/4.0/).

Volume & Issue: دوره 12 -

Title: تأثیر انتقال دانش پنهان بر شایستگی بالینی پرستاران

http://rehabilitationj.uswr.ac.ir/article-1-1020-fa.html

هدف:این پژوهش با هدف تعیین تأثیر انتقال دانش پنهان به روش داستان‌گویی بر شایستگی بالینی پرستاران آی. سی. یو. بیمارستان فاطمه زهرا شهر بوشهر در سال 1390 انجام شده است. روش بررسی: این مطالعه پژوهشی نیمه تجربی از نوع قبل و بعد است که بر روی 16 نفر از پرستاران شاغل در بخش آی. سی. یو که به صورت نمونه‌گیری مبتنی بر هدف انتخاب شدند انجام شده است.جامعه پژوهش شامل کل پرستاران شاغل در بیمارستان فاطمه زهرا (41 نفر) بودند. ابزار پژوهش یک پرسشنامه خود ابرازی مشتمل بر مهارتهای عمومی‌و تخصصی شایستگی بالینی بود. 4 جلسه داستان‌گویی برای انتقال تجارب برای نمونه‌ها برگزار شد و پرسشنامه‌ها قبل و بعد از مداخله توسط شرکت کنندگان تکمیل و نتایج در پایان با استفاده از آزمون تی زوجی تحلیل شد. یافته‌ها: در بررسی شایستگی بالینی به صورت کلی میانگین نمرات پیش آزمون 219/50 و میانگین نمرات پس‌آزمون 239/69 بوده است که میانگین نمرات پس‌آزمون به طور معناداری بیشتر از پیش‌آزمون است (0/003=P). نتیجه‌گیری: نتایج حاصل از این پژوهش نشان می‌دهد که انتقال تجارب و دانش از طریق داستان‌گویی باعث ارتقاء شایستگی بالینی پرستاران در زمینه‌های مختلف مربوط به شایستگی بالینی می‌شود.

Title: تعیین پایایی بین والدین و درمانگران نسخه فارسی سیستم طبقه‌بندی توانایی دستی (MACS) برای کودکان فلج مغزی

http://rehabilitationj.uswr.ac.ir/article-1-1022-fa.html

هدف: تعیین پایایی بین والدین و درمانگران نسخه فارسی سیستم طبقه‌بندی توانایی دستی (MACS) برای کودکان فلج مغزی(CP) می‌باشد. روش بررسی: جهت تعیین پایایی بین والدین و درمانگران نسخه فارسی MACS، بعد از انجام مراحل ترجمه، ترجمه بالعکس و روایی محتوا، پایایی تکرار آزمون و پایایی بین ارزیابان در میان والدین و کاردرمانگران با استفاده از ضریب همبستگی درون خوشه ای (ICC) و ضریب کاپای وزنی مورد ارزیابی قرار گرفت. 100 کودک 18-4 سال با میانگین سنی ۸۵/15 بر حسب ماه؛ 37 دختر و 63 پسر با انواع مختلف فلج مغزی مورد بررسی قرار گرفتند. یافته‌ها: پایایی بین ارزیابان عالی بود؛ ضریب همبستگی درون خوشه ای (ICC) بین والدین و کاردرمانگران در نوبت اول (۰٫96) و در نوبت دوم (۰/95) و بالای ۰/9 به دست آمد و ضریب کاپای وزنی نیز بالای ۰/75بوده که هر دو نشان دهنده میزان توافق عالی است. برای پایایی تکرار آزمون ICC در مورد والدین (۰/97) و هم درمانگران (۰/98) بالای ۹۰ به دست آمد و همچنین ضریب کاپای وزنی نیز بالای ۰/75 بوده که هر دو بیانگر میزان همبستگی بالا بوده‌اند. میزان توافق کلی بین دو سیستم MACS و GMFCS به دست آمد. نتیجه گیری. نسخه فارسی مقیاس MACS از پایایی بین والدین و درمانگران کافی برخوردار است و می‌توان از آن به عنوان ابزاری مفید و کاربردی در ارزیابی میزان توانایی دستی کودکان فلج مغزی استفاده نمود.

Title: مطالعه موردی تأثیر ارتز داینامیکی نئوپرنی بر پارامترهای موثر بر راه رفتن یک کودک فلج مغزی اسپاستیک دای پلژی

http://rehabilitationj.uswr.ac.ir/article-1-1033-fa.html

هدف: پژوهش حاضر با هدف بررسی تأثیر ارتز داینامیکی نئوپرنی بر پارامترهای کینماتیکی موثر بر راه رفتن در یک کودک فلج مغزی اسپاستیک دای پلژی و همچنین جهت برطرف نمودن نواقص موجود در طراحی و ساخت این ارتز جدید و گشودن عرصه تحقیقات بیشتر در این زمینه می‌باشد. روش بررسی: این مطالعه گزارش موردی و به لحاظ هدف کاربردی می‌باشد. تحقیق بر روی یک دختر ۱۱ ساله مبتلا به فلج مغزی اسپاستیک دای پلژی با الگوی راه رفتن کراچ انجام گرفت. بعد از ساخت ارتز مطابق با اندازه های کودک، ارزیابی اولیه با استفاده از دستگاه آنالیز حرکت ۶ دوربینه (Vicon 460) انجام شد و داده های کینماتیکی و پارامترهای فضایی- زمانی ثبت شدند. ارزیابی‌های ثانویه، بعد از پوشیدن ارتز به مدت ۶ هفته هر روز ۴ تا ۵ ساعت نیز انجام شد و تغییرات زوایای مفاصل هیپ، زانو ومچ با و بدون ارتز ارزیابی شدند. یافته‌ها: نتایج این مطالعه در مورد تأثیر بلافاصله‌ایی ارتز در ابتدای مطالعه، کاهش بیشتر از 5 درجه را در حداکثر و حداقل فلکشن زانو و دامنه حرکتی مفصل هیپ، با ارتز نشان داد و تعداد گام در دقیقه با ارتز افزایش یافت. در مورد تأثیر بلافاصله ایی با ارتز بعد از 6 هفته، حداکثر و حداقل فلکشن هیپ و حداقل فلکشن زانو بیشتر از 5 درجه کاهش یافت و طول گام حدودا 12 سانتی متر افزایش یافته است. همچنین بعد از 6 هفته مداخله، در حالت بدون ارتز، میانگین حداکثر فلکشن زانو، دامنه حرکتی مفصل هیپ و زانو بیشتر از 5 درجه کاهش یافتند و تعداد گام در دقیقه افزایش یافت.  نتیجه‌گیری: با استفاده از ارتز داینامیکی نئوپرنی راستای بیومکانیکی اندام تحتانی به سمت بهبودی پیش رفت و الگوهای حرکتی مفاصل نیز روان‌تر شدند. همچنین در این مطالعه با توجه به این که ارتز فقط اندام تحتانی را دربرمی‌گرفت، مشکلات مطالعات قبلی تا حدودی رفع گردید.

Title: بررسی مشکلات رفتاری در کودکان فلج مغزی

http://rehabilitationj.uswr.ac.ir/article-1-1041-fa.html

هدف: مشکلات رفتاری طیف وسیعی از مشکلات دوران کودکی را تشکیل می‌دهد. کسب اطلاعات در زمینه وسعت مشکلات رفتاری در کودکان فلج مغزی به منظور شناسایی و مدیریت این مشکلات و ارتقای خدمات روانشناختی در زمینه سلامت روانی کودکان فلج مغزی ضروری است.هدف از این مطالعه بررسی مشکلات رفتاری در کودکان فلج مغزی است. روش بررسی: در مطالعه مقطعی حاضر، 90 کودک فلج مغزی 12-4 ساله مراجعه کننده به مراکز توانبخشی شهر تهران در سال 1390-1389، به روش در دسترس انتخاب شدند. ابزار اندازه گیری مشکلات رفتاری در این پژوهش، نسخه والدی پرسشنامه توانایی‌ها و مشکلات بود که دارای 5 خرده مقیاس است. اطلاعات جمع‌آوری شده با کمک جداول توزیع فراوانی، شاخص‌های مرکزی و پراکندگی، توصیف و با استفاده از تست‌های آماری کای-دو و رگرسیون تحلیل گردید. یافته‌ها: نتایج نشان داد که میزان شیوع مشکلات رفتاری در کودکان فلج مغزی 43/3 درصد می‌باشد. هم چنین نتایج میزان شیوع برحسب زیرگروه‌های طبقات تشخیصی مشکلات رفتاری پرسشنامه توانایی‌ها و مشکلات نشان داد که مشکل در روابط با همسالان(٪82/2) بیشترین و مشکلات بیش فعالی (٪4/4) کمترین میزان شیوع را داشتند. هم چنین بین نمره کلی مشکلات رفتاری با سطح عملکرد حرکتی درشت و بهره هوشی کودک رابطه آماری معناداری وجود دارد (0/05>P). نتیجه‌گیری: با توجه به اینکه درصد بالایی از کودکان فلج مغزی، مشکلات رفتاری دارند، برنامه‌های آموزشی و مداخلات روانشناختی پیشنهاد می‌شود.

Title: مقایسه فرسودگی شغلی و اختلالات اسکلتی عضلانی در بین کاربران رایانه و کارکنان اداری

http://rehabilitationj.uswr.ac.ir/article-1-1052-fa.html

هدف: فشارهای روانی ،افزایش بروز اختلالات اسکلتی عضلانی ،بی تحریکی و خستگی از عوارض سوء گسترش فناوری علی رغم افزایش تولید و بهره‌وری می‌باشند .هدف این پژوهش تعیین فرسودگی شغلی و اختلالات اسکلتی-عضلانی در کاربران کامپیوتر و دیگر کارکنان اداری غیره کار بر ،مقایسه این دو گروه و ارائه پیشنهاد جهت کنترل عوامل خطر در صورت وجود چنین مشکلاتی در محیط کار روش بررسی: یک پژوهش مقطعی که بر روی 426 نفر از کارکنان اداری (40 درصد مرد و 60 درصد زن) که بر اساس میزان استفاده از رایانه در هفته، به دو گروه کاربر و غیره کاربر تقسیم شدند و فرسودگی شغلی و اختلالات اسکلتی عضلانی بین این دو گروه مقایسه شده است. یافته‌ها: یافته‌ها نشان داد که در کل کارکنان بیشترین میزان فرسودگی شغلی مربوط به کاهش کیفیت فردی 29٫2درصد کمترین میزان مربوط به مسخ شخصیت 4٫6درصد می‌باشد، بر اساس آزمون من ویتنی در سه بعد فرسودگی شغلی هیچ اختلاف معناداری بین گروه کاربر رایانه و غیره کاربر وجود نداشت اما بین دو گروه در میزان اختلالات اسکلتی عضلانی در گردن (0/03=P)، شانه راست (0/03=P)، پشت (0/044=P)، مچ دست راست ( 0/03=P) اختلاف معنادار وجود دارد به طوری که در گروه کاربر رایانه میزان اختلالات بیشتر می‌باشد. نتیجه‌گیری: بر اساس نتایج، میزان فرسودگی در بعد کاهش کیفیت فردی و همچنین میزان دردهای کمری و پشتی در کارکنان اداری نسبتاً بالا می‌باشد که مستلزم ارائه راهکارهای ارگونومی جهت بهبود کیفیت زندگی کاری و جلوگیری از پیشرفت عوامل ایجاد اختلالات اسکلتی عضلانی می‌باشد.

Title: بررسی شیوع اختلالات اندام فوقانی در فیزیوتراپیست‌های شهر تهران

http://rehabilitationj.uswr.ac.ir/article-1-1055-fa.html

هدف: اختلالات مرتبط با کار بخش مهمی از بیماری‌های اسکلتی عضلانی را تشکیل می‌دهد. فیزیوتراپیست‌ها به دلیل کاربرد زیاد دست در حین کار در معرض اختلالات مختلف به ویژه مشکلات مرتبط با اندام فوقانی قرار دارند. هدف از این طرح بررسی میزان شیوع این اختلالات در فیزیوتراپیست‌های شهر تهران بوده است. روش بررسی: پرسشنامه‌ای حاوی اطلاعات زمینه‌ای، شغلی و اختلالات احتمالی اندام فوقانی و نوع آن‌ها تهیه و در اختیار تعدادی از فیزیوتراپیست‌های شهر تهران به صورت تصادفی قرار گرفت. که نهایتا اطلاعات ۹۶ نفر که کامل بود وارد پروسه تحقیق شد. یافته‌ها: تعداد قابل ملاحظه‌ای از آن‌ها دارای مشکلات اندام فوقانی بودند به طوری که ۷۴ درصد مردان و ۸۹ درصد زنان دارای حداقل یک اختلال در اندام فوقانی خود بودند. با افزایش سابقه کار بالینی، میزان شیوع این مشکلات بیشتر می‌شد. شایع‌ترین درگیری و آسیب در شانه و در مرحله بعد در مچ دست بوده است. شدت درد در معیار VAS در مردان بیشترین فراوانی متعلق به نمره 5 و 4 بوده و در زنان، 3، 4، 6 و 8 بوده است. نتیجه‌گیری: شیوع مشکلات اندام فوقانی در فیزیوتراپیست‌ها بالاست و لذا نیازمند توجه جدی جهت کاهش این مشکلات و افزایش کارایی فیزیوتراپیست‌ها می‌باشد.

Title: بررسی ارتباط شاخص توده بدنی و کنترل پوسچرال سالمندان

http://rehabilitationj.uswr.ac.ir/article-1-1018-fa.html

هدف: زمین خوردن در جمعیت در حال رشد سالمندان، مشکل شایعی است. تحقیقات نشان می‌دهد اختلال تعادل و کنترل پاسچرال نقش عمده ای در زمین خوردن سالمندان دارد. از سوی دیگر شواهد بیومکانیکی نشان می‌دهد که تعادل پوسچرال با افزایش شاخص توده بدنی بدتر می‌شود. در پژوهش‌های انجام شده به گروه سنی بالای 60 سال توجهی نشده است. این پژوهش با هدف تعیین یکی از عوامل مؤثر در زمین خوردن سالمندان و همچنین پیشنهاد روش برای پیشگیری از این خطر انجام شده است. روش بررسی: در یک پژوهش مقطعی 30 زن و مرد بالای 60 سال انتخاب شدند. میزان نمره آزمون برگ و شاخص توده بدنی در آن‌ها اندازه‌گیری شد و رابطه بین آن بررسی گردید. یافته‌ها: یافته‌ها نشان داد که ارتباط بین شاخص توده بدنی و تعادل بر اساس آزمون پیرسون ۰/39 =R و معنادار (۰/05>P) بود. رابطه‌ای بین سن و تعادل دیده نشد ولی تعادل در مردان بهتر از زنان بود. نتیجه‌گیری: به نظر می‌رسد که بین شاخص توده بدنی و تعادل سالمندان ارتباط وجود دارد و این شاخص می‌تواند به عنوانی معیاری برای پیش‌بینی زمین خوردن در سالمندان استفاده گردد.

Title: بررسی تکرارپذیری ارزیابی الکترومیوگرافی فعالیت عضلات اکستانسور ناحیه کمری-لگنی در حین الگوی حرکتی اکستانسیون ران در وضعیت دمر در ورزشکاران مبتلا به سابقه استرین همسترینگ

http://rehabilitationj.uswr.ac.ir/article-1-1019-fa.html

هدف: در این تحقیق به بررسی تکرارپذیری ارزیابی الکترومیوگرافی (EMG) عضلات ناحیه کمری-لگنی در حین اکستانسیون ران در وضعیت دمر در ورزشکاران با استرین مزمن همسترینگ پرداختیم. روش بررسی: یک نمونه 10 نفری از ورزشکاران با سابقه استرین همسترینگ مورد مطالعه قرار گرفتند. سیگنال آمپلی تود EMG عضلات ارکتور اسپاین، گلوتئوس ماگزیموس، باسپس فموریس و سمی‌تندینوسوس در طول اکستانسیون ران در وضعیت دمر 3 بار اندازه‌گیری شد. آزمون‌های ICC و SEM برای ارزیابی تکرارپذیری نسبی و مطلق استفاده شدند. یافته‌ها: شاخص تکرارپذیری نسبی برای تمام عضلات ارزیابی شده بیشتر از ٪90 بود. نتایج تکرارپذیری بالایی را برای فعالیت EMG عضلات ناحیه کمری-لگنی در حین اکستانسیون ران در وضعیت دمر در ورزشکاران با استرین مزمن همسترینگ نشان می‌دهند. نتیجه‌گیری: تست اکستانسیون ران در وضعیت دمر می‌تواند از تکرارپذیری خوبی برای ارزیابی الگوی فعالیت عضلات کمری-لگنی در افراد با سابقه استرین همسترینگ برخوردار است.

Title: مقایسه حرکات چرخشی کتف بین بیماران مبتلا به سندروم گیرافتادگی شانه و افراد سالم حین بالابردن بازو در صفحه اسکاپولا با استفاده از مارکر آکرومیون کلاستر

http://rehabilitationj.uswr.ac.ir/article-1-1021-fa.html

هدف: هدف این مطالعه، مقایسه حرکات چرخشی کتف با استفاده از مارکر آکرومیون کلاستر و دستگاه آنالیز حرکتی وایکون حین بالابردن بازو در صفحات اسکاپولا بین بیماران مبتلا به سندروم گیرافتادگی شانه و افراد سالم است. روش بررسی: این تحقیق یک مطالعه پایه‌ای و کاربردی است که به مقایسه تفاوت حرکات چرخشی ثبت شده کتف با مارکر آکرومیون کلاستر بین 15 فرد مبتلا به سندروم گیرافتادگی شانه (میانگین سنی ۱۴/۲۴۲±۴۶/۶، 7 مرد و 8 زن) و 13 فرد سالم (میانگین سنی ۱۴/۳۰۹±۴۷/۴۶۱، 7 مرد و 6 زن) که از لحاظ سن، قد و وزن با گروه بیمار تطابق داشته‌اند، پرداخته است. در نهایت از آزمون‌ تی و نسخه 18 نرم‌افزار SPSS مورد استفاده قرار گرفت.   یافته‌ها: کلیه مشخصات زمینه‌ای بیماران توزیعی نرمال داشت. بنابر اطلاعات ثبت شده، الگوی حرکات چرخشی کتف در هر دو گروه سالم و بیمار مشابه بود اما میزان درجه چرخش رو به بالای کتف در گروه مبتلا به طور قابل توجهی کمتر از گروه سالم مشاهده شد (۰٫۴۸=P) نتیجه‌گیری: می‌توان بیان داشت که حرکات چرخشی کتف نقش مهمی‌در ریتم حرکتی گلنومرال دارند که در ارزیابی و درمان بیماران مبتلا به سندروم گیرافتادگی شانه باید مدنظر قرار بگیرد، از طرف دیگر مارکر آکرومیون کلاستر ابزار مناسبی جهت ثبت حرکات کتف به خصوص حرکات چرخش رو به بالای کتف با دستگاه آنالیز حرکتی می‌باشد.

Title: رابطه هوش معنوی با راهبردهای مقابله با استرس

http://rehabilitationj.uswr.ac.ir/article-1-1028-fa.html

هدف: پژوهش حاضر با هدف بررسی رابطه‌ی هوش معنوی بر راهبردهای مقابله‌ با استرس در دانشجویان انجام گرفت. روش بررسی: در این مطالعه‌ی توصیفی-همبستگی جامعه‌ی‌ مورد پژوهش شامل دانشجویان دانشگاه‌ علوم بهزیستی و توانبخشی است. تعداد 150 نفر از دانشجویان به‌ روش نمونه‌گیری تصادفی طبقه‌ای انتخاب شدند.ابزار جمع آوری داده‌ها در این مطالعه‌ شامل پرسشنامه‌ی‌ هوش معنوی کینگ و مقیاس راهبردهای مقابله ‌با استرس بوده‌ است. داده‌ها توسط نرم افزار SPSS17 مورد تحلیل قرار گرفت و از آزمون های تی تست مستقل و رگرسیو خطی ساده و آمار توصیفی برای این منظور استفاده شد.  یافته‌ها: توسط آمار توصیفی میانگین و انحراف معیار هوش معنوی و راهبردهای مقابله با استرس دانشجویان محاسبه شد و توسط آزمون تی مستقل تفاوت نمرات دختران و پسران در دو متغیر هوش معنوی و راهبردهای مقابله با استرس معنادار (۰/۰۰۱>P) بدست آمد.همچنین تحلیل رگرسیون نشان می‌دهد رابطه‌ی معنادار (۰/۰۰۱>P) میان دو متغیر هوش معنوی و راهبردهای مقابله با استرس وجود دارد. نتیجه‌گیری: میان هوش معنوی و راهبردهای مقابله با استرس رابطه معنادار(۰/۰۰۱>P) وجود دارد وهوش معنوی می‌تواند پیش‌بینی کننده‌ی‌ راهبردهای مقابله با استرس باشد.
